# Minimally invasive perineal redo surgery for rectovesical and rectovaginal fistulae: A case series

**DOI:** 10.1016/j.ijscr.2020.11.067

**Published:** 2020-11-19

**Authors:** A.A.J. Grüter, S.E. Van Oostendorp, L.J.H. Smits, M. Kusters, M. Özer, J.A. Nieuwenhuijzen, J.B. Tuynman

**Affiliations:** a Amsterdam UMC, Vrije Universiteit Amsterdam, Department of Surgery, Cancer Center Amsterdam, De Boelelaan 1117, Amsterdam, Netherlands; b Amsterdam UMC, Vrije Universiteit Amsterdam, Department of Plastic Surgery, Cancer Center Amsterdam, De Boelelaan 1117, Amsterdam, Netherlands; c Amsterdam UMC, Vrije Universiteit Amsterdam, Department of Urology, Cancer Center Amsterdam, De Boelelaan 1117, Amsterdam, Netherlands

**Keywords:** Rectovesical fistula, Rectovaginal fistula, SILS, Minimally invasive, Perineal redo surgery, Case series

## Abstract

**Introduction:**

Iatrogenic recto-urogenital fistulae are refractory complications that rarely heal without surgical intervention. The ongoing local infection causes pain, discomfort and substantially impacts quality of life. Surgical repair requires adequate exposure and space to fill with healthy tissue, which is a major challenge in pelvic redo surgery. An abdominal approach to repair the fistula is associated with major morbidity and often fails to expose the deep pelvis. In our experience a novel transperineal minimally invasive approach a utilizing single incision laparoscopic surgery (SILS) technique could offer improved results.

**Presentation of cases:**

In the present study, three cases of patients with recto-urogenital fistulae after pelvic surgery are described. Two patients were diagnosed with a rectovesical fistula and one patient with a rectovaginal fistula. In all three cases, a minimally invasive perineal approach, using a SILS port, was used to perform surgical repair. The closure of the fistulae involved: a separate repair of the urethra/bladder or vaginal defect and the rectal defect, followed by interposition of vascularized tissue by either a pudendal thigh fasciocutaneous flap or omentoplasty.

**Discussion and conclusion:**

This study is the first to report on a minimally invasive perineal approach, utilizing a SILS technique for recto-urogenital fistulae repair after previous pelvic surgery. The current approach improves exposure, creates surgical space, optimizes view and allows the interposition of vascularized tissue, without causing substantial blood loss and avoiding major abdominal surgery.

## Highlights

• Iatrogenic recto-urogenital fistulae rarely heal without surgical treatment.
• The major challenge in pelvic redo surgery is to achieve adequate exposure.
• A minimally invasive perineal approach using a SILS port seems a good treatment.

## 1 Introduction

Pelvic surgery is associated with local complications. These complications occur particularly in patients who underwent previous pelvic radiation. Persisting recto-urogenital fistulae occur in 0–10%, are often resistant to conservative therapy and substantially impact patients’ quality of life [1], [2], [3]. A fistula is defined as an abnormal connection between two epithelialized surfaces such as skin, blood vessel, bowel lumen or hollow organ (bladder, gallbladder). Anorectal fistulae represent a large share of problems in the field of coloproctology with an incidence of 0.021% [4]. In a minority of patients recto-urethral/vesical or rectovaginal fistula can occur, which are most frequently caused by surgery, trauma, diverticulitis, Crohn’s disease and cancer [5], [6]. In particular the iatrogenic rectovaginal or rectovesical fistulae after pelvic surgery rarely heal without surgical reintervention [7], [8]. Definitive surgical treatment of the fistula is associated with postoperative morbidity, such as surgical site infections (22%), urethral incontinence (16.7%), intra-abdominal abscesses (16.7%), as well as a high failure rate i.e. recurrence of the fistula (44%) [9], [10]. Currently, there is no consensus about the optimal method of surgical repair. The major challenge in pelvic redo surgery is to achieve adequate exposure. The recto-urethral/vesical and rectovaginal fistulae are located anteriorly, deep and low in the pelvis, where the surgical approach is hampered by distortion of the anatomy due to extensive fibrosis, caused by previous surgery and the preceding septic episode.

The standard abdominal approach often requires full mobilisation of the omentum and re-adhesiolysis, which is associated with conversion to open surgery. Transanal Minimally Invasive Surgery (TAMIS) is a less invasive approach, which is slowly being implemented as a perineal approach to pelvic surgery. The spectrum of pathology that can be managed with TAMIS has evolved from excision of small rectal lesions to a transanal total mesorectal excision (TaTME), salvage surgery in anastomotic leakages, treatment of endometriosis and pouch surgery [11], [12]. It is also applicable in transvaginal right hemicolectomy with natural orifice specimen extraction (NOSE) [13].

A transperineal approach using a single incision laparoscopic surgery (SILS) port might be better suited for surgical treatment of fistulae located distally in the pelvis. This approach could offer improved access with good exposure compared to the conventional transabdominal approach. In this single-centre retrospective study, we report three cases of minimally invasive perineal redo surgery using a SILS port for the surgical treatment of recto-urogenital fistulae. The aim of this study is to provide a detailed description of this surgical approach and to evaluate this procedure.

This study was conducted in accordance with the modified PROCESS criteria, which provides a structure for reporting surgical case series [14].

## 2 Presentation of case 1

**Fig. 1:**
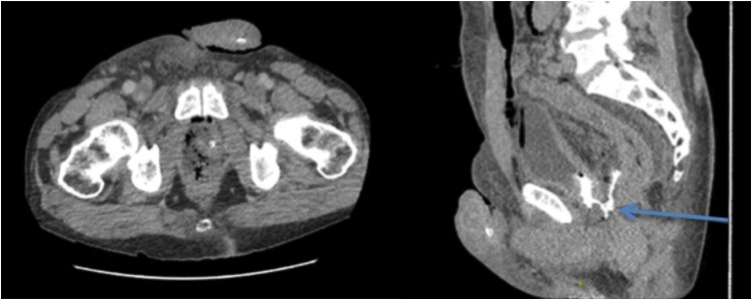
CT abdomen with intravesical contrast administered via catheter, illustrates a small amount of contrast in the urinary bladder with extraluminal contrast collected in the prostate bed. This indicates a dorsal bladder wall defect, suggesting that there is an open communication between the urinary bladder and surgical bed. The arrow points to the extraluminal contrast. On the left side (transverse section), a defect of the ventral rectal wall is illustrated.

A 60-year-old male developed a rectovesical fistula after a robot-assisted radical prostatectomy (RARP) for a pT2cN0M0 prostate adenocarcinoma. The five year relative survival rate in this stage of the disease is estimated on 100%. On postoperative day (POD) 2 urinary leakage from the rectum, obvious complaints of a urinary tract infection and the results of the CT abdomen (*see* Fig. 1) indicated the presence of a rectovesical fistula. As initial treatment a fecal diverting transversostomy was constructed and bilateral nephrostomy catheters were inserted for urinary diversion. Besides, the initial operative treatment was combined with antibiotic treatment.

The diverting fecal and urine interventions successfully decreased the ongoing inflammation, but failed to heal the fistula. Three months after constructing the ostomy, a definitive surgical repair was performed in a joint operation by a team of an urologist, a plastic surgeon and a colorectal surgeon. A minimally invasive perineal approach was performed by using a SILS port applied in a transverse skin incision in the perineum. The first step of the procedure included exploration and dissection through the perineal body, exposing the fistula tract. The dissection was continued proximal to the fistula tract up to the peritoneal fold, avoiding entry of the abdomen. This step is performed to optimize the placement of the future flap. The next step included extraluminal closure of the vesicourethral anastomosis (first layer). Afterwards the rectal defect was closed from the perineal-retroperitoneal space with a V-Loc running suture. Simultaneously, a pudendal thigh fasciocutaneous flap of the left groin was mobilized, pedicled on the external pudendal artery and deepithelialized (*see* Fig. 4). This flap was inserted between the closed vesical wall and the rectum and sutured proximal to the defects to reduce the possibility of recurrence (second layer). Finally, the defect in the wall of the rectum was again closed transanally by a two-row running suture (third layer) (*see* Box 1 *for a detailed step by step description*). The procedure was completed without perioperative complications and negligible blood loss (<50cc). The total duration of surgery from incision to final closure was 317 min.

Box 1 **Step by step procedure for rectovesical fistula closure (see video).**

**Table:** 

Step 1:	Identify the fistula tract using a transanal approach using a SILS port. The tract is excised and the rectal wall is separated from the urethra and vesical wall.
Step 2:	Closure of the urethral-vesical anastomosis defect with sutures transanally. Alternatively, this can be done transperineally after full dissection of the space between the rectum and bladder.
Step 3:	Make a perineal incision and position the SILS port. The minimally invasive perineal approach is used to further dissect the rectal wall from the vesical wall.
Step 4:	Create a pudendal thigh fasciocutaneous flap of the left groin. Mobilize the flap, pedicle it on the external pudendal artery and deepithelialized it. After the flap is deepithelialized, insert the pudendal thigh flap into the perineal wound with guidance of the camera to allow optimal placement across the defect of the previous fistula.
Step 5:	Position and suture the flap proximally to both rectal and vesical defects. The transanal approach with a SILS port is used to position and suture the flap.
Step 6:	The rectal wall is closed with sutures transanally with the use of a SILS port.

Alt-text: Box 1

Postoperatively, intravenous antibiotic therapy (cefazolin and metronidazole) was continued for 5 days. Diversion was continued to facilitate unprovoked healing of the operation area. Repeated CT scans with rectal contrast at six and eight months of follow-up were performed to assess the presence of any potential recurrence or inflammation at the site of the previous fistula tract. After clinical and radiological confirmation of a healed fistula, the nephrostomy catheters and the transversostomy could be reversed eight months after the initial treatment. The patient recovered uneventful and has not shown any signs of fistula recurrence up until 6 months after reversal.

## 3 Presentation of case 2

**Fig. 2:**
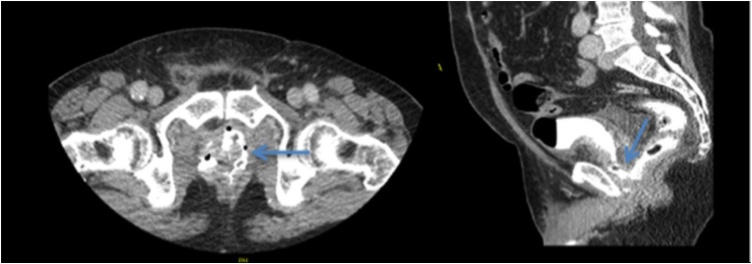
CT abdomen with intravescial contrast administered via catheter, shows a connection between the urinary bladder and the rectum, illustrating the rectovesical fistula. The arrow points out the rectovesical fistula.

A 77-year-old male patient underwent a robot-assisted radical prostatectomy (RARP) for a pT2cN0M0 prostate adenocarcinoma. The estimated 5-year relative survival of this patient nearly 100%. On POD 7, leakage of urine from the rectum and fecally contaminated urine indicated the presence of a rectovesical fistula. A CT abdomen with intravesical contrast administered via catheter confirmed a rectovesical fistula (*see* Fig. 2). To control the sepsis a sigmoidostomy was constructed for fecal diversion and a transurethral catheter was inserted for urinary diversion.

Four months after the initial procedure, both the defects of the vesical wall and the rectal wall were closed transanally. One week after the procedure a new transurethral catheter was replaced due to luxation. Unfortunately, one day after the luxation of the catheter, the patient experienced urinary leakage from the rectum caused by a recurrence of the fistula tract.

Six months after the initial procedure, a new attempt to surgically repair the fistula tract was performed by a multidisciplinary surgical team containing an urologist, plastic surgeon and colorectal surgeon. The minimally invasive perineal approach was applied using the SILS technique as is utilized in TAMIS. The procedure included a debridement of the fistula tract, closure of the defects of the vesical wall (first layer) and the rectal wall (third layer) and an interposition of a pudendal thigh flap (*see* Fig. 4) (second layer) to reduce the possibility of recurrence (*see* Box 1 *for a detailed step by step description*). The duration of the procedure was 189 min. No perioperative difficulties or complications were observed and the estimated amount of blood loss was minimal (<40cc).

After the operation, antibiotics (cefuroxime and metronidazole) were continued for 5 days. No signs of recurrence of the rectovesical fistula were found during the immediate postoperative course. The fistula tract resolved, which was objectified during a cystography at six months of follow-up. Hereafter, the urinary catheter could be removed. The patient is in training to improve his continence of spontaneous micturition at the urologic outpatient clinic and is on the waiting list for colostomy reversal.

## 4 Presentation of case 3

**Fig. 3:**
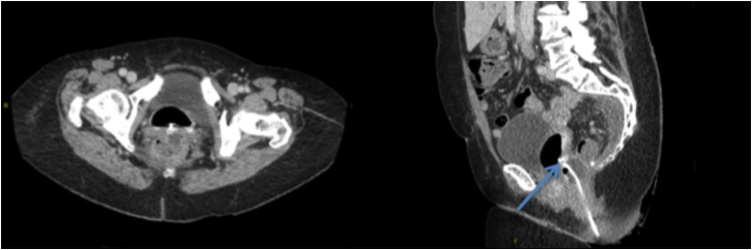
CT scan of the abdomen with rectal contrast shows a rectovaginal fistula. The arrow points to the rectovaginal fistula.

A 65-year-old woman underwent a completion TaTME after previous local excision of a high-risk pT1 adenocarcinoma of the rectum (well-to-moderately differentiated, Kikuchi level sm3, no lymphatic and vascular invasion with clear resection margins) with an estimated survival rate of 90%. On POD 12, vaginal passage of gas and stool indicated the presence of a rectovaginal fistula. A CT-scan of the abdomen confirmed a rectovaginal fistula (*see* Fig. 3).

**Fig. 4:**
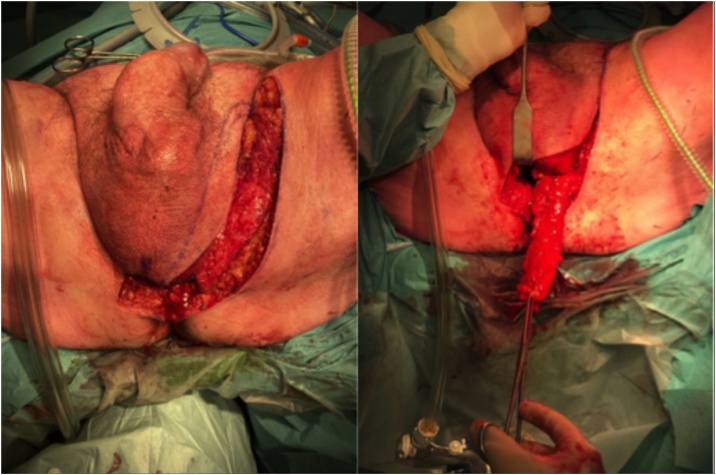
Perineal incision with the pudendal thigh flap.

A diverting loop ileostomy was constructed for fecal diversion and the colon was antegradely flushed to discard feces. Hereafter, but during the same operation, a SILS port was positioned for both transvaginal and transanal minimal invasive surgery. Again, the first step included exploration and dissection of the fistula. Secondly, a transperineal approach was utilized to dissect the space between rectum and vagina both proximal and distal to allow optimal mobilization of rectum and vagina to close the defect without tension. The next step consisted of suturing the vaginal wall. Simultaneously an omentoplasty was performed which facilitated the insertion of the mobilized omentum up to the perineal body and was closed subcutaneously. Finally, the defect in the wall of the rectum was closed by a two-row running suture; hence again resulting in a three-layer closure (*see* Box 2 *for a detailed step by step description*). The total surgery time of this operation was 285 min. The blood loss during this procedure was less than 30cc and so no blood transfusions were needed.

Box 2 **Step by step procedure for rectovaginal fistula closure.**

**Table:** 

Step 1:	Laparoscopic mobilization of an omentum in order to facilitate an omentoplasty.
Step 2:	Identification of the fistula tract by a transanal approach. Excision of the fistula tract traced and separation of the rectal and vaginal wall.
Step 3:	Transperineal access with small incision to create more space distally and proximally of the fistula using a SILS port.
Step 4:	Sutured closure of the vaginal wall from transvaginal placement of the port.
Step 5:	Bring down the omentoplasty to the perineum bridging the site of the previous fistula tract. Transanal anchoring sutures of the omentoplasty subcutaneously.
Step 6:	Transanal full thickness closure of the defect in the rectal wall with the use of a SILS port.
Step 7:	Reversed leak test by filling the pelvic cavity from the abdominal site with saline. Insufflation and transanal and transvaginal inspection of the closure sites for air leakage.

Alt-text: Box 2

Antibiotic therapy (oral ciproxin and intravenous metronidazole) was continued for 5 days. After the interventions, no recurrence of the fistula was noticed during the follow-up examinations. A CT scan with rectal contrast at 2 months of follow-up was performed to evaluate potential recurrence or inflammation at the site of the earlier fistula tract. After clinical and radiological confirmation that the area was healed, without any signs of recurrence, the ileostomy could be reversed five months after the initial surgical treatment. After the reversal, the patient recovered and has not shown any signs of fistula recurrence up until 6 months after reversal.

## 5 Discussion

Recto-urogenital fistulae are refractory complications following pelvic surgery. Redo surgery, especially for the most distal fistulae, is challenging because of complex pelvic adhesions. Besides, it brings a large burden to patients as a result of low success rate and associated morbidity. Therefore, it is of major importance to cure fistulae during the first attempt.

The transperineal technique is a very old approach utilized by the very first surgeons, but this technique was hampered by poor vision, small operation field and poor control of blood loss. With the utilization of the modern insufflation, the SILS technique and a HD camera, the exposure is maximized with minimal invasiveness. In the cases described above, the minimally invasive perineal approach with the use of a SILS port was applied. Upon diagnosis, fecal and urinary diversion must be started immediately by a nephrostomy, ileostomy or colostomy in order to control the ongoing pelvic sepsis [15]. This type of initial therapy is recommended for a period of three to six months [16], [17]. After the “cooling-off” period, persisting fistulae should proceed to operative repair. In our experience the minimally invasive perineal approach provides an excellent view and exposure of the described distal recto-urogenital fistulae after previous pelvic surgery. In addition, it avoids major abdominal surgery. On the other hand, laparoscopic abdominal approach is recommended for high rectovaginal and sigmoidvaginal fistulae. Van der Hagen et al. showed a high healing percentage of 95% after laparoscopic abdominal repair [18]. However, abdominal access to urogenital fistulae deep in the pelvis is often a major challenge, since inflammation and fibrosis turns this area into a “no-man’s land”, with obliteration of surgical planes which may sequel to high peri-operative morbidity [19].

Various interposition flaps have been used in the last decades and these interpositions could be the key to surgical success to repair the fistulae. The endorectal advancement flap procedure uses a partial-thickness flap of rectal wall to cover the defect in the rectovaginal septum and was recommended as the treatment of choice for patients with distal rectovaginal fistulae in the early 1980s. The initial results were very promising with healing rates ranging between 78 and 95% [20]. However, later on, patients who had been treated with an endorectal advancement flap showed relatively high recurrence rates of 57% [21]. The puborectal sling interposition for treatment of rectovaginal fistulae has been described with a success rate of 62% [22]. For the rectovesical fistulae, an array of interposition flaps have been investigated in the past as well, including flaps of the gluteus maximus, omentum, gracilis, dartos and levator ani muscle [15]. Ghoniem et al. reported 25 patients with a rectovesical fistula that developed after treatment for prostate cancer (radical prostatectomy, radiotherapy, cryotherapy or combination therapy) treated with transperineal approach and a gracilis muscle interposition. None of the 25 participants had a recurrence with a mean follow-up of 28 months [23].

In the future, additional studies are necessary to assess the efficacy and safety of this minimally invasive perineal approach using a SILS port. In patients with distal recto-urogenital fistulae after previous pelvic surgery, the outcomes of this new technique need to be compared with the conventional abdominal approach. Since recto-urogenital fistulae are a complex problem and tailored approach on an individual patient level is required, a randomized controlled trial is unlikely to be feasible and case-matched studies will conceivably be suited to provide further insight in optimizing treatment strategies. Nevertheless, extensive prospective research may provide long-term outcomes of the described procedure and may identify patients who could benefit most.

## 6 Conclusion

A new minimally invasive perineal approach using a SILS port is proposed in pelvic redo surgery for recto-urogenital fistulae in the most distal part of the pelvis. Usage of the described technique provides potential benefits in visualization and exposure. The three-layer repair using well vascularized tissue like an omentum slip or a pudendal thigh fasciocutaneous flap may lead to lower recurrence rates.

## Declaration of Competing Interest

The authors report no declarations of interest.

## Funding

This research did not receive any specific grant from funding agencies in the public, commercial, or not-for-profit sectors.

## Ethics approval

Waived by ethical committee of Amsterdam UMC, location VUmc.

## Consent

Patient signed informed consent for the use of clinical data, images of CT scan and video of surgical procedure.

## Author contribution

A.A.J. Grüter wrote the paper. S.E. Van Oostendorp, L.J.H. Smits, M. Kusters, M. Özer, J.A. Nieuwenhuijzen and J.B. Tuynman gave their feedback on the paper. J.B. Tuynman, M. Özer and J.A. Nieuwenhuijzen performed the surgical procedure.

## Registration of research studies

This work is registered in Research Registry with the unique identifying number: researchregistry6219.

## Guarantor

J.B. Tuynman.

## Provenance and peer review

Not commissioned, externally peer-reviewed.

## CRediT authorship contribution statement

**A.A.J. Grüter:** Investigation, Methodology, Writing - original draft, Resources, Visualization. **S.E. Van Oostendorp:** Supervision, Writing - review & editing. **L.J.H. Smits:** Writing - review & editing. **M. Kusters:** Writing - review & editing. **M. Özer:** Writing - review & editing. **J.A. Nieuwenhuijzen:** Writing - review & editing. **J.B. Tuynman:** Conceptualization, Writing - review & editing.
